# Missing and accounted for: gaps and areas of wealth in the public health review literature

**DOI:** 10.1186/1471-2458-11-757

**Published:** 2011-10-03

**Authors:** Daiva Tirilis, Heather Husson, Kara DeCorby, Maureen Dobbins

**Affiliations:** 1 Faculty of Health Sciences, McMaster University, 1200 Main St. W., Hamilton, Ontario, Canada

## Abstract

**Background:**

High-quality review evidence is useful for informing and influencing public health policy and practice decisions. However, certain topic areas lack representation in terms of the quantity and quality of review literature available. The objectives of this paper are to identify the quantity, as well as quality, of review-level evidence available on the effectiveness of public health interventions for public health decision makers.

**Methods:**

Searches conducted on http://www.health-evidence.ca produced an inventory of public health review literature in 21 topic areas. Gaps and areas of wealth in the review literature, as well as the proportion of reviews rated methodologically strong, moderate, or weak were identified. The top 10 topic areas of interest for registered users and visitors of http://www.health-evidence.ca were extracted from user profile data and Google Analytics.

**Results:**

Registered users' top three interests included: 1) healthy communities, 2) chronic diseases, and 3) nutrition. The top three preferences for visitors included: 1) chronic diseases, 2) physical activity, and 3) addiction/substance use. All of the topic areas with many (301+) available reviews were of interest to registered users and/or visitors (mental health, physical activity, addiction/substance use, adolescent health, child health, nutrition, adult health, and chronic diseases). Conversely, the majority of registered users and/or visitors did not have preference for topic areas with few (≤ 150) available reviews (food safety and inspection, dental health, environmental health) with the exception of social determinants of health and healthy communities. Across registered users' and visitors' topic areas of preference, 80.2% of the reviews were of well-done methodological quality, with 43.5% of reviews having a strong quality rating and 36.7% a moderate review quality rating.

**Conclusions:**

In topic areas in which many reviews are available, higher level syntheses are needed to guide policy and practice. For other topic areas with few reviews, it is necessary to determine whether primary study evidence exists, or is needed, so that reviews can be conducted in the future. Considering that less than half of the reviews available on http://www.health-evidence.ca are of strong methodological quality, the quality of the review-level evidence needs to improve across the range of public health topic areas.

## Background

### Using Systematic Reviews

A systematic review consists of an examination of all of the primary studies on a topic, which includes searching for, collating, and assessing the studies, to establish conclusive evidence about a topic [1]. The Cochrane Collaboration is an international body that produces systematic reviews of primary research at the highest standard, and as such, this is a commonly accepted definition of systematic reviews. Evidence-informed public health advocates the incorporation of the best available scientific evidence into decision making [2]. Review level evidence is an important part of evidence-informed public health decision making, since reviews synthesize the results of individual studies, providing a more accurate estimate of the effects of an intervention [3]. Rigorous synthesis of primary research minimizes bias [4-6], explains differences among studies relating to the same research question [7,8], and presents more precise and consistent summary statistics than the effect sizes found in individual studies [5,8-10]. Well-conducted reviews provide high-quality, accurate evidence [4-6], increasing decision-makers' confidence in the strength of the review evidence and in applying the findings in practice [4]. Public health decision makers prefer using systematic reviews to assist in decision-making given that review level evidence saves time and is more efficient compared to using primary studies [6,11]. Systematic review findings can be generalized to a larger sample, providing a great evidence base for users; such external validity is essential to ensure adaptability and applicability of evidence-based interventions into the local context [12]. Consequently, systematic reviews are useful for informing and influencing public health policy and practice decisions [7,13,14].

### Challenges in Public Health Decision Making

While the value of review level evidence is acknowledged and well documented, public health decision makers encounter a number of challenges in incorporating systematic reviews in their decision making.

#### Access

Review level evidence is available in journals and obtainable through bibliographic database searches [15], yet barriers still exist in accessing the information. Public health decision-makers often have difficulties locating systematic reviews in the published literature due to database indexing limitations, limited availability of relevant public health reviews, lack of primary study evidence and thus a lack of reviews in some topic areas, and a lack of interest in certain topic areas by researchers conducting reviews [3,15,16].

#### Relevance

Even when systematic reviews are identified, only a small proportion of those are relevant to public health. For example, one search strategy captured 41, 871 abstract titles across all research topics in public health but once screened, only 1, 356 were identified as being potentially relevant, of which only 207 reviews were actually deemed relevant to public health [16]. The majority of published systematic reviews pertain to clinical topics rather than public health [16]. Consequently, there are gaps at the systematic review level across the spectrum of public health practice [6,17].

#### Appraisal

When relevant reviews are located, users still need to be critical of that evidence. There are a number of search engines that provide evidence from various databases, such as the Cochrane Database of Systematic Reviews, PubMed, and the Campbell Collaboration, but the evidence is not critically appraised [17,18]. While some of these databases which include public health relevant evidence assess the quality of the evidence, many do not [19]. To reduce bias in evidence-informed practice, public health decision makers need to be able to assess the methodological quality of systematic reviews [4]. However, critical appraisal skills have been identified as a significant barrier to using research evidence in decision making [20]. Development of individual capacity is important in addressing appraisal challenges as well as providing support [21].

### Health Evidence: Supporting Public Health Decision Making

Health Evidence is a research and service organization aimed at supporting Canada's public health decision makers in accessing and interpreting research evidence. The target audience for Health Evidence includes medical officers of health, policy makers, program managers, and frontline workers in public health. Given the audience, decision making may take place at the local level (such as public health units/regional health authorities), provincial level (such as ministries), or federal level (such as government). Our most widely accessible resource is the http://www.health-evidence.ca online registry of systematic reviews; a free, user-friendly, searchable database of public health relevant, quality-appraised systematic reviews published since 1985 evaluating the effectiveness of public health interventions. Given that unpublished literature, such as conference abstracts, provide little added value [22], Health Evidence mainly focuses on published review literature. In order to identify the scope of interventions to include in the health-evidence.ca registry, qualitative interviews were conducted, as well as seeking organizational charts and information from every province and territory in Canada on the services public health units provide. Systematic reviews are considered relevant if: 1) the article is a review, which includes the synthesis of more than one primary study; 2) the intervention is relevant to public health practice; 3) the effectiveness of an intervention is evaluated; 4) the evidence on health outcomes is reported; and 5) the search strategy is described [19]. To assess the methodological quality, the following ten criteria are used: 1) a clearly focused question was stated; 2) inclusion criteria were explicitly stated; 3) a comprehensive search strategy was described; 4) an adequate number of years were covered in the search; 5) a description of the level of evidence was provided; 6) the methodological rigor of primary studies was conducted and results were described; 7) the methodological quality of primary studies was assessed by two reviewers and the level of agreement was provided; 8) tests of homogeneity or assessment of similarity of results across studies was conducted and reported; 9) appropriate weighting of primary studies was conducted; and 10) the author's interpretation of the results were supported by the data [19]. Each criterion is equally weighted and a final methodological score is tallied out of 10. Reviews with an overall rating of eight or more are considered strong, five to seven, moderate, and below four are considered to be weak in methodological quality.

Due to competing demands, it is necessary for decision makers to quickly find, assess and use evidence to inform their decision making. The health-evidence.ca registry eliminates the need for users to search individual databases, identify relevant reviews, and conduct critical appraisal on the effectiveness of public health interventions. The tools used by Health Evidence to assess relevance and conduct critical appraisal are available online http://health-evidence.ca/html/HowJudgeforYourself, accessed 6 May 2011), and users can view completed critical appraisal tools for each review in the registry.

In order to reach public health decision makers, Health Evidence is promoted at conferences, workshops, and site-visits, through outreach and engagement, networking, and listservs via website posts and e-newsletters, and through social media, such as Twitter and YouTube. Health Evidence also connects with public health decision makers through various partnerships and collaborations with the National Collaboration Centres for Public Health, public health units, the Canadian Best Practices Portal, and the Public Health Agency of Canada. The registry is also listed as a resource on several public health organization and university websites, such as Research into Action, Pan American Health Organization, KT+ Knowledge Translation, Canadian Institute for Health Research (CIHR) Knowledge Translation and Commercialization, Nova Southeastern University, Dalhousie University, and more.

Health-evidence.ca has nearly 5, 000 registered users, and sees over 40, 000 visitors annually representing more than 150 countries. In the development of the health-evidence.ca registry, 21 topic areas of interest to public health decision makers were identified through focus groups and consultations with key informants within the public health setting. The purpose of the registry is to facilitate access to review-level evidence for decision makers working in program planning and policymaking in public health and health promotion [19]. All reviews in the registry are indexed according to these 21 *Focus of Review *topic areas allowing site visitors to search the registry using common public health terms. In addition, each registered user completes a profile when signing up to the site checking off as many of the 21 topic areas relevant to them. This enables each registered user to receive a list of reviews related to their areas of interest, along with a rating of the methodological quality of each review, each quarter when the registry is updated.

Unfortunately, for some public health topics, there are limited or no high quality reviews available and for others the reviews that are available are not of good methodological quality, meaning that use of these findings in decision making requires careful consideration. A thorough search of http://www.health-evidence.ca allowed us to indentify the top areas of interest to public health decision makers, and provide an overview of the availability of review-level evidence within these areas. In this paper we will not only identify topic areas of high interest to public health decision makers, we will also highlight existing gaps as well as identify topic areas with an abundance of high-quality evidence. One objective of this paper is to identify the quantity of systematic reviews available on the effectiveness of public health interventions, so as to encourage researchers and research funders to conduct/fund systematic reviews where gaps exist. A second objective is to identify the quality of systematic review evidence on the effectiveness of public health interventions in order to encourage higher quality methodological reviews and higher level synthesis of topics areas rich in high-quality reviews (e.g., review of reviews).

## Methods

### Populating the health-evidence.ca registry of systematic reviews

The health-evidence.ca registry of systematic reviews is populated through an extensive ongoing search (1985-present) of seven electronic databases (MEDLINE, EMBASE, CINAHL, PsycINFO, Sociological Abstracts, BIOSIS, SportDiscus), handsearching of 46 journals, and screening the reference lists of all relevant reviews [19]. Reviews are assessed for relevance, and then relevant reviews are indexed by commonly-used public health terms and quality assessed by two independent reviewers who come to agreement on the final rating of each review (strong, moderate, weak). More detail on http://www.health-evidence.ca has previously been published [19].

### Assessing health-evidence.ca user and visitor areas of interest

Registered user areas of interest were assessed by querying the health-evidence.ca registered user database and looking at the areas of interest identified by all users who registered up to December 31, 2010. Data were aggregated by topic area. Registered user data is provided voluntarily by users and aggregation ensures individual data remain anonymous. Topic areas of interest were ranked from highest to lowest rates of user interest. The top 10 areas of interest were summed to generate the denominator: total user interest in the top 10 topic areas.

Visitor areas of interest were assessed by summing frequency of visitor searches of the 21 *Focus of Review *topic areas and visitor use of the topic area browse menu for the period January 1, 2010 to December 31, 2010. Visitor site usage is tracked via Google Analytics, a web analytics tool that collects and aggregates non-personal data to report on visitor interaction with the health-evidence.ca website. Total search and browse access by unique visitors were ranked from highest to lowest pageviews. The top 10 areas of interest were summed to generate the denominator: total visitor interest in the top 10 topic areas.

### Availability of public health review literature by user/visitor areas of interest

The health-evidence.ca registry was used to identify gaps and areas of wealth in the public health review literature. Each of the 21 Focus of Review topic areas were searched, and the quantity and proportion of reviews rated methodologically strong, moderate, and weak were identified. Three categories were used to define availability of reviews within each topic area: *(+) few*, representing 1-150 reviews; *(++) moderate*, representing 151-300 reviews; and, *(+++) many*, representing topic areas possessing greater than 301 reviews. Reviews that addressed multiple topics were accounted for within each topic area that they addressed (e.g., a review on the effectiveness of exercise in preventing chronic disease would be categorized as both physical activity and chronic disease).

## Results

### Top 10 Topic Areas of Interest

#### Top 10 registered users' areas of interest

**Figure 1:**
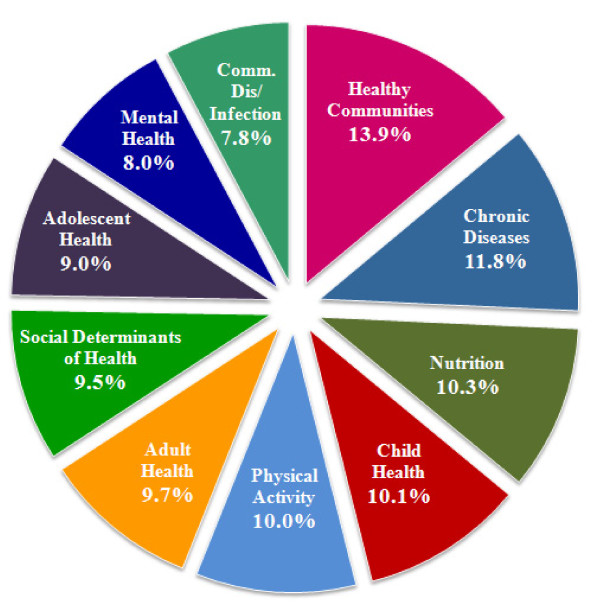
**Top 10 priority topic areas for http://www.health-evidence.ca registered users**. Percentage interest reflects the top 10 areas of interest; percentages add up to 100%

As of December 31, 2010, there were 4, 842 health-evidence.ca registered users, with each user identifying an average of 6.3 areas of interest, resulting in a total of 30, 363 identified topic areas. Upon registration, each user is asked to indicate as many areas of interest as they find relevant, which results in more identified areas of interest than total users. For the purpose of accurately representing the data showing all interest, we have included all indications in interest in each topic area, knowing that the denominator used represents total expressions of interest as opposed to total users. The top 10 registered users' topic areas are represented in Figure 1. Registered users' top three topic areas out of the top 10 include by order of interest: 1) healthy communities, 2) chronic diseases, and 3) nutrition.

#### Top 10 visitors' areas of interest

**Figure 2:**
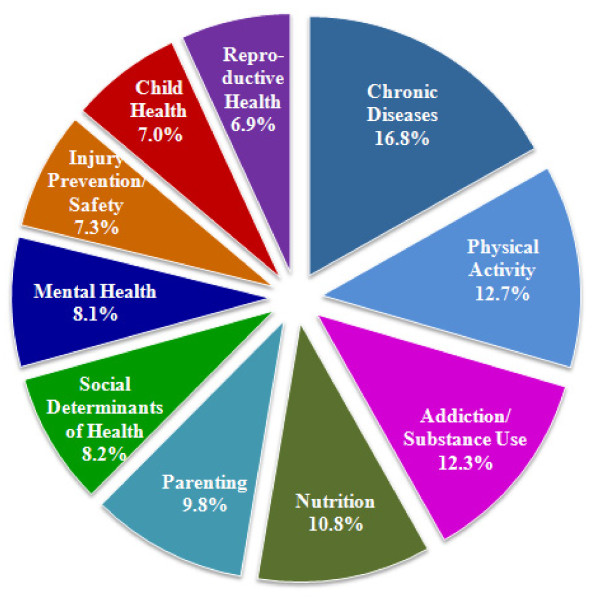
**Top 10 priority topic areas for visitors to http://www.health-evidence.ca**.

Between January 1, 2010 and December 31, 2010, health-evidence.ca had a total of 40, 166 visits to the website, representing 24, 593 unique visitors (15, 573/40, 166 visits were return visitors to the site). The average visitor spent just under four minutes on the site. Across this time span total visitors viewed 132, 259 unique pages, representing an average of 3.3 unique pageviews per visit. Of the pages viewed, 22, 774 were search results representing a search within the 21 *Focus of Review *topic areas and 3, 289 were pageviews representing a visitor accessing the topic area browse menu. Summed total pageviews for the 21 public health relevant topic areas on health-evidence.ca were 26, 063. These data do not include submission of free-text searches to the health-evidence.ca registry within this period. The top 10 visitors' topic areas of interest are represented in Figure 2. The top three preferences by order of interest include: 1) chronic diseases, 2) physical activity, and 3) addiction/substance use.

#### Comparing interest between registered users and visitors

The top 10 topic areas of interest of registered users and the top 10 topic areas of interest of visitors of http://www.health-evidence.ca, as well as the availability of review evidence by methodological quality, are identified in Table 1. The top areas of interest and the total number of reviews available included: addiction/substance use (355), adolescent health (367), adult health (552), child health (409), chronic diseases (702), communicable disease/infection (241), healthy communities (134), injury prevention/safety (296), mental health (336), nutrition (426), parenting (287), physical activity (353), reproductive health (240), and social determinants of health (66).

**Table 1:** Overview of registered user's and visitors' top 10 preferred topic areas and the availability of review evidence by methodological quality

Reg. Users' Top 10	Visitors' Top 10	Main Topic Areas	TOTAL	Strong	Moderate	Weak
**1**	-	Healthy Communities	**134**	*49*	*50*	*35*
**2**	1	Chronic Diseases	**702**	*286*	*261*	*155*
**3**	4	Nutrition	**426**	*190*	*149*	*87*
**4**	9	Child Health	**409**	*161*	*160*	*88*
**5**	2	Physical Activity	**353**	*136*	*129*	*88*
**6**	-	Adult Health	**552**	*248*	*200*	*104*
**7**	6	Social Determinants of Health	**66**	*31*	*25*	*10*
**8**	-	Adolescent Health	**367**	*145*	*133*	*89*
**9**	7	Mental Health	**336**	*141*	*133*	*62*
**10**	-	Communicable Disease/Infection	**241**	*116*	*81*	*44*
**-**	3	Addiction/Substance Use	**355**	*127*	*136*	*92*
**-**	8	Injury Prevention/Safety	**296**	*147*	*106*	*43*
**-**	5	Parenting	**287**	*135*	*99*	*53*
**-**	10	Reproductive Health	**240**	*121*	*74*	*45*

While there was overlap between six of the registered users' and visitors' top areas of interest, the topic areas healthy communities, adult health, adolescent health, and, communicable disease/infection were preferred by registered users alone, and visitors had preferences for addiction/substance use, parenting, injury prevention/safety, and, reproductive health.

For the six areas of interest similar to both registered users and visitors, differences exist in the order of expressed interest. Three topic areas ranked similarly among the top 10 for both registered users and visitors with a difference of only one rank apart: chronic disease ranked high on both top 10 lists, ranking first for visitors and second for registered users; nutrition ranked third for registered users and fourth for visitors; and social determinants of health ranked sixth for visitors and seventh for registered users. The remaining three topic areas that both visitors and registered users were interested in included: mental health, ranking seventh for visitors and ninth for registered users; physical activity, ranking second for visitors and fifth for registered users, and, child health, ranking fourth for registered users and ninth for visitors. In the top five highest ranking common topic areas, both groups had interest in chronic diseases, nutrition, and, physical activity

Characteristics of registered users of and visitors to the Health Evidence registry are provided in Table 2. Based on the sample, 82.7% of registered users and 65.0% of visitors to health-evidence.ca are Canadian, and English is the language of preference for 98.1% of registered users and 83.3% of visitors. Upon registering to health-evidence.ca, users are asked to provide their organizational affiliation; 54.6% of users work in the field of public health or health services. As of December 31, 2010, 94.8% of registered users were subscribers to the quarterly Health Evidence tailored e-newsletter.

**Table 2:** Characteristics of registered users and visitors from January 1 to December 31, 2010

	Registered Users (4, 668) % (n)	Visitors (40, 166) % (n)
**Continent**		

**North America**	90.6% (4, 229)	75.0% (30, 107)
Canada	82.7% (3, 859)	65.0% (26, 113)
*British Columbia*	*10.9% (509)*	*4.6% (1, 841)*
*Alberta*	*7.8% (366)*	*4.6% (1, 853)*
*Saskatchewan*	*3.6% (166)*	*1.6% (627)*
*Manitoba*	*4.0% (186)*	*1.9% (761)*
*Ontario*	*41.6% (1940)*	*41.9% (16, 810)*
*Quebec*	*5.3% (247)*	*6.0% (2, 408)*
*New Brunswick*	*2.2% (105)*	*0.7% (282)*
*Nova Scotia*	*3.4% (157)*	*2.2% (900)*
*Prince Edward Island*	*0.3% (12)*	*0.2% (88)*
*Newfoundland & Labrador*	*1.8% (82)*	*0.9% (354)*
*Nunavut*	*0.5% (22)*	*0.1% (29)*
*Northwest Territories*	*0.3% (16)*	*0.1% (49)*
*Yukon*	*0.3% (15)*	*0.2% (77)*
*Not specified (within Canada)*	*0.8% (36)*	*0.1% (34)*
United States of America	7.9% (370)	9.9% (3, 994)
**Europe**	3.5% (164)	14.4% (5, 774)
**Oceania**	4.0% (188)	4.0% (1, 604)
**Asia**	0.7% (35)	3.9% (1, 570)
**South America, Central America, Caribbean**	0.7% (31)	1.9% (777)
**Africa**	0.1% (6)	0.8% (326)
**Not specified**	0.3% (15)	0.02% (8)

**Language Preference**^**§**^		

English	98.1% (4, 580)	83.3% (33, 459)
French	1.9% (88)	6.2% (2, 482)
Spanish	-	3.6% (1, 463)
Italian	-	1.8% (708)
German	-	1.0% (399)
Portuguese	-	0.7% (285)
Other	-	3.4% (1, 370)

Organization Type*		

Public Health/Health Services	54.6% (2, 547)	15.8% (5, 502)
Academic/Research Institution	18.7% (872)	18.3% (6, 393)
Not-for-Profit/NGO	4.5% (210)	0.4% (123)
Government	4.2% (197)	6.7% (2, 340)
Corporate/Private Business	2.0% (93)	0.8% (294)
Professional Association	0.9% (44)	0.1% (49)
Library	0.6% (28)	0.8% (287)
School (primary/secondary)	0.2% (10)	0.01% (5)
Pharmaceutical	0.1% (6)	0.01% (5)
Internet Provider	0.04% (2)	53.0% (18, 503)^†^
Not specified/determinable	14.1% (659)	4.0% (1, 408)

^§ ^Language preference data only collected for Canada's official languages (English and French) for registered users, whereas 65 languages were identified for visitors.

* Visitor data for organization type is shown for organizations with five or more visits in 2010.

^† ^Visitor data was collected by internet service provider and many organizations could only be identified by internet service provider (e.g. Rogers, Shaw, Bell, etc.)

### Availability of Review Evidence

**Figure 3:**
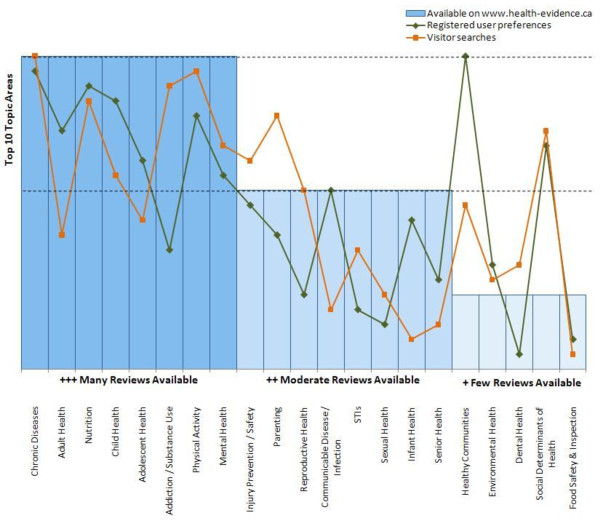
**Availability of reviews, registered users' preferences and visitor searches**.

As of April 1, 2011 there were 2, 175 systematic reviews evaluating the effectiveness of public health and health promotion interventions indexed in the health-evidence.ca registry. Table 3 provides an overview of the availability of reviews within each of the 21 *Focus of Review *topic areas. Figure 3 depicts the relationship between registered users' interests, visitor searches, and available reviews within each of the 21 topic areas.

**Table 3:** Comparison of the top ten priority topic areas by registered users and visitors and the availability of reviews

Main Topic Areas	Review Availability	Registered Users	Visitors
Addiction/Substance Use	+++		X
Adolescent Health	+++	X	
Adult Health	+++	X	
Child Health	+++	X	X
Chronic Diseases	+++	X	X
Communicable Disease/Infection	++	X	
Dental Health	+		
Environmental Health	+		
Food Safety & Inspection	+		
Healthy Communities	+	X	
Infant Health	++		
Injury Prevention/Safety	++		X
Mental Health	+++	X	X
Nutrition	+++	X	X
Parenting	++		X
Physical Activity	+++	X	X
Reproductive Health	++		X
Senior Health	++		
Sexual Health	++		
Sexually Transmitted Infections	++		
Social Determinants of Health	+	X	X

(+)few reviews as 1-150, (++)moderate reviews as 151-300, (+++)many reviews as 301 and greater

Topic areas with fewer than 150 reviews included: food safety and inspection (13), dental health (62), social determinants of health (66), environmental health (69), and healthy communities (134). Of the areas with fewer than 150 reviews, healthy communities ranked first as an area of interest for registered users with 2, 548 registered users indicating interest in this topic. Social determinants of health ranked sixth and seventh for visitors and registered users respectively, with 1, 528 visitor searches submitted in 2010 and 1, 734 registered users indicating interest in the topic. The majority of health-evidence.ca registered users did not have preference for the other three identified areas with fewer than 150 reviews.

Topic areas with a moderate number of reviews (151-300 reviews) included: infant health (153), senior health (152), sexual health (195), sexually transmitted infections (208), communicable disease/infection (229), reproductive health (232), parenting (282), and, injury prevention/safety (241). Four of these eight topic areas have expressed interest by visitors or registered users. For visitors, ranking at fifth, eighth and tenth place respectively, searches submitted in 2010 for parenting totalled 1, 813 pageviews, for injury prevention/safety 1, 360, and, for reproductive health 1, 273 pageviews. Ranking tenth place, 1, 422 registered users indicated interest in the topic communicable disease/infection.

Topic areas with a large quantity of systematic reviews (301 or more reviews) include: mental health (336), physical activity (353), addiction/substance use (355), adolescent health (367), child health (409), nutrition (426), adult health (552), and chronic diseases (702). All of the topic areas with many available reviews (301+) were of interest to registered users and/or visitors. Chronic diseases, nutrition, and, physical activity were the three highest-ranking areas of interest common across both groups. The number one visitor area of interest and number two registered user area of interest was chronic diseases with 3, 115 visitor searches submitted in 2010, and 2, 153 registered users expressing interest in the topic. Nutrition ranked third for registered users and fourth for visitors with 1, 877 user interests, and 2, 010 visitor searches respectively. Physical activity ranked second for visitors with 2, 350 searches, and fifth for registered users with 1, 824 interested in the topic. Child health and mental health were two additional topics with many available reviews that rank in the top ten for both registered users and visitors. In the area of child health, 1, 846 registered users expressed interest and 1, 293 visitors submitted searches, ranking it fourth and ninth respectively, and the area of mental health ranked seventh for visitors with 1, 502 searches submitted, and ninth for registered users with 1, 459 interested in the topic. The remaining three topic areas with many available reviews were preferred by either visitors or registered users (i.e., not common to both groups). Addiction/substance use ranked third for visitors with 2, 283 search page views in 2010. Adult health and adolescent health ranked sixth and eighth respectively for registered users with 1, 778 and 1, 636 registered users indicating interest in each of these topics.

A master list categorizing all of the 21 topic areas and the corresponding number of reviews available on the effectiveness of public health interventions, as well as the methodological quality, are listed in the additional file 1.

The 21 *Focus of Review *topic areas were further broken down into 291 sub-topic categories. There were 34 sub-topics with no reviews available including: hormone replacement therapy, infertility, Norwalk virus, autism, and elder abuse, among others (Table 4). The 21 *Focus of Review *topic areas that had sub-topics with no review included adult health, communicable disease/infection, dental health, environmental health, food safety and inspection, parenting, and senior health. The largest proportion of sub-topic with no review was observed within communicable disease/infection (n = 12). Adult health was ranked sixth and communicable disease/infection ranked tenth by registered users. Parenting was ranked as a fifth preference for visitors. The remaining sub-topic with no reviews within dental health, environmental health, food safety and inspection, and senior health were not a preference for either registered users or visitors.

**Table 4:** Overview of sub-topic areas with no review evidence (N = 34)

Adult Health
Women's Health
Female genital mutilation
Hormone replacement therapy
Hysterectomy
Infertility
Communicable Disease/Infection
Food borne diseases
Hand foot mouth disease
Hantavirus
Heliobacter pylori
Impetigo
Norwalk virus
Pink eye/conjunctivitis
Pinworm
Ringworm
Sexually transmitted infections
Tapeworm
Toxic shock syndrome
Dental Health
Dental implants
Environmental Health
Asbestos
Carbon monoxide poisoning
Environmental epidemiology
Extreme temperature
Flood
Radiation exposure
Swimming pool
Food Safety & Inspection
Botulism
Bovine spongiform encephalopathy
Fish borne
Food processing & inspection
Hepatitis A
Shellfish borne
Parenting
Autism
Senior Health
Elder abuse
Urinary incontinence

In addition there were 68 sub-topics with fewer than five reviews available, such as lung cancer, testicular cancer, food service inspection, fetal alcohol syndrome, sexual assault, and social justice. The full list of sub-topics with fewer than five reviews is included in Table 5. Most of the sub-topics with fewer than five reviews were within the registered users and visitors' topic areas of interest. Topic areas which were of interest to both registered user and visitors only had a small proportion of sub-topics with less than five reviews available (child health, chronic diseases, mental health, nutrition, and social determinants of health). Whereas the communicable disease/infection topic area, which ranked tenth among registered users, had the largest proportion of sub-topics with fewer than five reviews (n = 16). While environmental health and food safety and inspection were not preferred topic areas for registered users and visitors, a large portion of the sub-topics in these two categories had fewer than 5 reviews. Although there is currently a lack of review literature in these areas, these topics have been mentioned by public health professionals as relevant to public-health practice.

**Table 5:** Overview of sub-topic areas with less than 5 reviews available (N = 68)

Addiction/Substance Use
Gambling
Solvent abuse
Adult Health
Men's Health
Partner violence
Prostate cancer
Testicular cancer
Child Health
Ear infection
Speech language
Vision
Chronic Diseases
Arthritis
Cancer
Lung cancer
Oral cancer
Testicular cancer
Communicable Disease/Infection
Avian flu
Blood borne diseases
Diphtheria
Head lice/pediculosis
Lyme disease
Pertussis
Poliomyelitis
Rabies
Rotavirus
SARS
Scabies
Streptococcal infection
Toxoplasmosis
Viral infections
West nile virus
Zoonoses
Environmental Health
Air pollution
ETS (environmental tobacco smoke)
Bioterrorism
Chemical exposure
Chemical safety
Electromagnetic fields
Environmental pollution
Hazardous waste
Insecticides
Lead poisoning
Mould
Noise exposure
Pest management
Pesticides
Sewage disposal
Water pollution
Food Safety & Inspection
E. coli
Fish borne
Food borne outbreaks
Food contamination
Food handling
Food poisoning
Food premise inspection
Food processing & inspection
Food safety
Food service inspection
Hepatitis A
Salmonella
Healthy Communities
Community development
Community health centers
Emergency preparedness
Injury Prevention/Safety
Air bags
Carpal tunnel
Drowning
Mental Health
Borderline personality disorder
Nutrition
Eating disorder
Binge eating
Parenting
Allergies
Reproductive Health
Fetal alcohol syndrome
Sexual Health
LGBT
Sexual assault
Social Determinants of Health
Food security/insecurity
Social justice

Also, there were numerous sub-topics with a great number of reviews available. Table 6 identifies 71 sub-topics with more than 25 reviews available. Such sub-topics included, but were not limited to, alcohol abuse/use, smoking cessation, women's health, cancer, cardiovascular disease, lifestyle behaviours, disease transmission, depression, diet, healthy weight, exercise, and HIV. As well, most of these sub-topics were within the registered users and visitors' topic areas of interest, with the exception of dental health, senior health, sexual health, and sexually transmitted infections.

**Table 6:** Overview of sub-topic areas with greater than 25 reviews available (N = 71)

Addiction/Substance Use
Alcohol abuse/use
Drug abuse/use
Smoking cessation
Tobacco use
Adult Health
Women's Health
Workplace health
Chronic Diseases
Cancer
Breast cancer
Cardiovascular disease
Blood pressure
Hypertension
Dementia/Alzheimer's disease
Diabetes
Lifestyle behaviours
Alcohol
Nutrition
Physical activity
Tobacco use
Obesity
Osteoporosis
Communicable Disease/Infection
Bacterial infections
Disease transmission
Influenza
Dental Health
Dental caries
Fluoride
Oral health
Healthy Communities
Community health services
Community wellness
Multicultural health
Injury Prevention/Safety
Falls
Injury
Safety
Violence
Mental Health
Anxiety disorders
Behaviour disorder
Depression
Mood disorder
Schizophrenia
Stress
Nutrition
Diet
Eating behaviour
Food intake
Fruit OR vegetables
Healthy weight
Supplements
Parenting
Child development
Child growth
Family functioning
Maternal child health
Parenting
Postnatal care
Physical Activity
Active living
Exercise
Health behaviour
Healthy weight
Lifestyle
Obesity prevention
Physical fitness
Reproductive Health
Breastfeeding
Maternal health
Pregnancy
Prenatal care
Prenatal health
Senior Health
Falls
Sexual Health
Pregnancy Prevention
Contraception
Condom use
Sexual behaviour
Sexually Transmitted Infections
AIDS
HIV
Social Determinants of Health
Income and Social Status

### Methodological Quality

The Health Evidence methodological quality rating is based on the ten criteria used to assess the strength of the methods. The proportion of reviews rated as having strong, moderate, or weak methodological quality was constant across all topic areas. 80.2% of the reviews on health-evidence.ca were of strong (43.5%) or moderate (36.7%) methodological quality. These well-done reviews included slightly more strong review quality ratings compared to the moderate review ratings. The remaining 19.8% of reviews in the top areas of interest were of weak methodological quality. These weak quality reviews met four or fewer methodological quality criteria, and as such, scored poorly on six or more of the ten criteria. Based on the weak reviews, 10.5% did not have a clearly focused question, 46.5% did not use appropriate inclusion criteria, 87.7% did not have a comprehensive search strategy, 34% did not cover an adequate number of years, 48% did not describe the level of evidence in the primary studies, 96.6% did not assess the methodological quality of the primary studies, 97% did not have transparent results, 53.9% did not appropriately combine the findings of the results across studies, 92.9% did not use appropriate methods to combine or compare results across studies, and 82.4% did not have data to support the author's interpretations.

## Discussion

A number of sub-topic areas within public health featured no reviews or a very small number of reviews including those within the main topic areas for women's health (sub-topics include female genital mutilation, hormone replacement therapy, and infertility as examples); communicable disease/infection (sub-topics include food-borne diseases, hantavirus, and Norwalk, among others); food safety and inspection (sub-topics include botulism, food processing and inspection, and Hepatitis A as examples); dental health (sub-topic: dental implants); environmental health (sub-topics include asbestos, carbon monoxide poisoning, environmental epidemiology, extreme temperature, swimming pools, and flood, as examples). In these areas, review literature is needed to add to the existing body of literature from which decision makers can draw. In topic areas lacking in review literature, realist reviews, which provide explanatory analyses [23], are a relatively new type of review that may provide further insight into the topic area.

While these areas show the deficits in review literature, in 25 other sub-topic areas there was a wealth of systematic review literature of moderate or strong quality, including but not limited to, alcohol abuse/use, smoking cessation, women's health, cancer, cardiovascular disease, lifestyle behaviours, disease transmission, depression, diet, healthy weight, exercise, and HIV. In these areas offering many reviews, most had 15 or more which were of strong methodological quality. While review groups can identify and fill gaps in areas where evidence is lacking, there is also an opportunity to produce higher level syntheses where good-quality review evidence is available. Based on this analysis of the published, public health review literature catalogued in http://www.health-evidence.ca, all of the topic areas having many available reviews were also preferred areas of interest for users of the site. It is unclear how the availability of evidence is linked to interest in a topic, but it may be that demand can generate reviews in a particular topic area and that the public spurs research to fill gaps [24]. Alternately, preference for a topic may be a reflection of there being available evidence that drives interest in the topic and web site updates regarding that topic. Interestingly, the 15 priority topic areas indicated by a 2005 Cochrane global priority setting exercise [24] still closely match the top 10 topic areas of interest indicated by health-evidence.ca users and visitors.

While resources such as http://www.health-evidence.ca, provide synthesized, high quality research evidence relevant to public health practice, coverage of health topics is not equal [25]. Compared to clinical review literature, there is far less population health systematic review literature [6] and within public health, a number of topic areas are without a solid base of review evidence evaluating the effectiveness of interventions (e.g., environmental health, social determinants of health) [18,26,27]. Public health studies are difficult to design and results are drawn from natural experiments (e.g. one health unit adopting a program compared to another health unit), thus fewer studies have been developed on the effectiveness of public health interventions compared to randomized controlled trials on medical treatments [28,29]. However this lack of review-level evidence doesn't necessarily indicate a lack of evidence [30]. There may be primary research or other forms of evidence that can inform decisions but which may not yet have been synthesized. In cases where there are no eligible studies available to be reviewed for a particular topic, the result can be an "empty review", meaning that no studies were located which met the inclusion criteria to answer the question for review. Empty reviews can go unreported but may in fact be useful since empty reviews indicate interest in an area, highlight gaps, and offer a snapshot of the state of research evidence at the time of publication [31]. Even in light of a lack of evidence, or poor reporting of the evidence, decision makers can and should take an informed approach to having insufficient evidence [30].

An informed approach may be needed in topic areas that demonstrated a lack of review-level evidence, such as dental health, environmental health, food safety and inspection, and seniors' health, and particularly for public health priority areas such as healthy communities and social determinants of health. Consequently, the best literature may be sparse or of low quality in these particular topic areas. In these areas, new systematic review literature is needed to inform practice and policy decision making. It is unclear at this time whether gaps pertain only to reviews lacking in these topic areas, or whether there is a corresponding lack of primary studies as well, hindering the production of reviews. In some instances it may be necessary to first develop the primary study base in order for studies to be available for synthesis in a systematic review. Future reviews should be conducted on these broad topic areas for which review-level evidence on the effectiveness of interventions is lacking. Funding organizations should generate calls for syntheses to address these gap areas, while non-government and public health organizations should provide feedback on the lack of evidence in areas of interest to them to potential funders. Funding priorities for syntheses should reflect those areas which are priorities for public health decision makers both in Canada and internationally.

There are promising indicators of demand for reviews, including actions being taken to promote the use of reviews [6,11,19,30,32-36], an awareness of sites providing access to review-level evidence [19] and an increasing number of groups generating summaries of reviews [33]. Despite this heightened activity, given the gaps, a greater investment is needed to provide an evidence base that can meet demand and determine how to apply existing good quality systematic reviews in different contexts [25]. Organizations involved in the conduct of systematic reviews should direct synthesis funding to areas lacking in review content, or should consider higher-level reviews of reviews (where appropriate), where large bodies of review evidence already exist. Examples of organizations that conduct systematic reviews and reviews of reviews include: The Cochrane Database of Systematic Reviews http://www.cochrane.org/reviews/index.htm, The Campbell Collaboration http://www.campbellcollaboration.org, The Centre for Reviews and Dissemination (CRD) http://www.york.ac.uk/inst/crd/index_databases.htm, Health Technology Assessment international (HTAi) http://www.htai.org/, Effective Public Health Practice Project (EPHPP) http://www.ephpp.ca, CDC Guide to Community Preventative Services http://www.thecommunityguide.org, Canadian Agency for Drugs and Technology in Health (CADTH) http://www.cadth.ca/, Agency of Healthcare Research and Quality (AHRQ) http://www.ahrq.gov/ and the National Institute for Health and Clinical Excellence (NICE) http://www.nice.org.uk. Some groups have a prioritization process to identify and meet needs for systematic reviews; for example, the Agency of Healthcare Research and Quality has a topic prioritization group to determine the relative importance of their effectiveness reviews against a standard set of criteria to prioritize unmet needs [37]. The Cochrane Collaboration aims to increase the quantity and quality of public health systematic reviews specifically [32], with the new Health Promotion and Public Health Review Group announced in 2006 to prioritize and produce public health relevant reviews [38]. Authors have suggested that a global registry of anticipated public health studies could help to fill the gaps by making it easier to identify relevant, but potentially unpublished, primary studies available for review [6].

While well-done reviews in a large number of areas are available, it is important to continue to improve the quality of the overall body of public health review literature. Considering that the majority of weak reviews scored poorly on assessing the methodological quality of the primary studies, transparency, methods for combining or comparing results, conducting a comprehensive search strategy, and data supporting the author's interpretations, review authors should be cognisant of these criteria when conducting systematic reviews. In improving the quality of systematic reviews, the overall goals should ensure there are high quality reviews in all public health topic areas and a higher standard across the board.

Although this paper provides information on the quantity and quality of systematic reviews on the effectiveness of public health interventions, there are a few limitations, including the lack of information regarding visitors to the Health Evidence registry. While the majority of visitors access the registry from various internet service providers, we are unable to determine their organization and the relevance of the registry content to a particular organization. Additionally, it is unclear at this time whether visitors and registered users are using reviews housed in the registry to inform their practice decisions. Studies are ongoing to evaluate the registry's usefulness and effectiveness.

## Conclusions

The result of this analysis demonstrates significant variation in the availability of review evidence across the 21 public health topic areas. For some topic areas, many reviews are available and a higher level synthesis is needed in order to provide decision makers with greater clarity to guide policy and practice for addiction/substance use, adolescent health, child health, chronic diseases, mental health, nutrition, and physical activity. At the other extreme however, there are topic areas for which there are few reviews to guide practice. Specifically, social determinants of health and healthy communities were identified as areas of interest that are lacking in review evidence. In these situations, an assessment is required to determine whether primary study evidence exists to contribute to reviews, or whether the primary studies are needed so that reviews can be conducted at some point in the future. Although there are gaps in the review literature on the effectiveness of public health interventions, just under half of the reviews available on health-evidence.ca are of strong methodological quality, demonstrating that good quality review level literature on which to base many public health programs and policies exists. It is hoped that the results of this analysis will provide direction to funders of reviews and organizations involved in conducting systematic reviews as to priority topic areas for consideration.

## Competing interests

The authors declare that they have no competing interests.

## Authors' contributions

DT wrote the initial draft of the manuscript, produced the tables, and incorporated contributing author feedback into the paper. HH contributed to the methods section, collated site statistics, produced the figures, and contributed to all drafts of the manuscript. KD contributed to the results and discussion sections, and contributed to all drafts of the manuscript. MD conceived the idea, secured funding for this project, and provided feedback on all drafts. All authors read and approved the final manuscript.

## Pre-publication history

The pre-publication history for this paper can be accessed here:

http://www.biomedcentral.com/1471-2458/11/757/prepub

## Supplementary Material

Additional file 1 **Overview of the availability of reviews by topic area and methodological quality**. Click here for file
