# Supplementary material for: Missing and accounted for: gaps and areas of wealth in the public health review literature
Source: BMC Public Health. 2011 Oct 3;11:757. doi: 10.1186/1471-2458-11-757 (PMC3229615; doi:10.1186/1471-2458-11-757)
Supplement: Additional file 1 — Overview of the availability of reviews by topic area and methodological quality. [file 1471-2458-11-757-S1.DOC]

# Additional files

**Additional file 1 – Overview of the availability of reviews by topic area and methodological quality***

| **Main Topic Areas** | **TOTAL** | **Strong** | **Moderate** | **Weak** |
| --- | --- | --- | --- | --- |
| Addiction/Substance Use | **355** | *127* | *136* | *92* |
| [Adolescent Health](http://www.health-evidence.ca/saved_searches/run_search/241) | **367** | *145* | *133* | *89* |
| Adult Health | **552** | *248* | *200* | *104* |
| [Child Health](http://www.health-evidence.ca/saved_searches/run_search/269) | **409** | *161* | *160* | *88* |
| Chronic Diseases | **702** | *286* | *261* | *155* |
| Communicable Disease/Infection | **241** | *116* | *81* | *44* |
| Dental Health | **62** | *36* | *16* | *10* |
| Environmental Health | **69** | *29* | *25* | *15* |
| Food Safety & Inspection | **13** | *7* | *4* | *2* |
| Healthy Communities | **134** | *49* | *50* | *35* |
| [Infant Health](http://www.health-evidence.ca/saved_searches/run_search/412) | **153** | *72* | *58* | *23* |
| Injury Prevention/Safety | **296** | *147* | *106* | *43* |
| Mental Health | **336** | *141* | *133* | *62* |
| Nutrition | **426** | *190* | *149* | *87* |
| Parenting | **287** | *135* | *99* | *53* |
| Physical Activity | **353** | *136* | *129* | *88* |
| Reproductive Health | **240** | *121* | *74* | *45* |
| Senior Health | **152** | *71* | *57* | *24* |
| Sexual Health | **195** | *78* | *76* | *41* |
| Sexually Transmitted Infections | **208** | *68* | *79* | *61* |
| Social Determinants of Health | **66** | *31* | *25* | *10* |

*Reviews addressing multiple topics are indexed within each topic area that they address. Total reviews by topic area do not add up to total reviews in the health-evidence.ca registry (n=2,175)
